# Supplementary material for: Controlling chaos using edge computing hardware
Source: Nat Commun. 2024 May 8;15:3886. doi: 10.1038/s41467-024-48133-3 (PMC11079072; doi:10.1038/s41467-024-48133-3)
Supplement: Supplementary file 1 — Supplementary Information [file 41467_2024_48133_MOESM1_ESM.pdf]

Supplementary Information

Controlling Chaos Using Edge Computing Hardware

Robert M. Kent,[1] Wendson A.S. Barbosa,[1] and Daniel J. Gauthier[1,2,*]

[1]The Ohio State University, Department of Physics, 191 West Woodruff Ave., Columbus, OH 43210, USA.

[2]ResCon Technologies, LLC, 1275 Kinnear Rd., Suite 239, Columbus, OH 43212, USA

*Correspondence to: gauthier.51@osu.edu

April 16, 2024

Supplementary Note 1: Comparing complexity with previous reservoir computing approach

In this section, we compare the complexity of the controller based on traditional reservoir computing by Canaday *et al.*[1] to our approach based on next-generation reservoir computing. We highlight three key metrics: the number of multiplications required to evaluate the control law which dominates the energy and space requirements on the FPGA, the number of tanh evaluations which requires look up tables, and the size of the total feature vector which affects the number of trainable parameters. Throughout this section, we use our notation to describe the traditional reservoir computing approach.

Inverse control with traditional reservoir computing

In the work by Canaday *et al.*,[1] evaluating the control law requires computing the updated reservoir state, which is analogous to computing the total feature vector $\mathbb{O}_{total,i} \in \mathbb{R}^N$ in our approach, then the reservoir state is mapped onto the control perturbation u_i used to drive the system to the desired state. We examine the mapping form of the reservoir state obtained from the differential form using an Euler integration method, as this is the form computed by the FPGA. The state of a reservoir with N nodes is given by

$$\mathbb{O}_{total,i} = \alpha \tanh\left(\mathbf{A}\mathbb{O}_{total,i-1} + \mathbf{W}_{in}^{x}\mathbf{X}_i + \mathbf{W}_{in}^{des}\mathbf{Y}_{des,i+m} + \mathbf{b}\right) + (1-\alpha)\,\mathbb{O}_{total,i-1}, \quad (1)$$

where $\alpha = \Delta t/c$, Δt is the timestep of the integration, c is the time constant, $\mathbf{A} \in \mathbb{R}^{N\times N}$ is the adjacency matrix determining the connections between nodes in the reservoir, $\mathbf{W}_{in}^{x} \in \mathbb{R}^{N\times d'}$ maps the accessible system variables $\mathbf{X}_i$ into the reservoir, $\mathbf{W}_{in}^{des} \in \mathbb{R}^{N\times d'}$ maps the desired state $\mathbf{Y}_{des,i+m} \in \mathbb{R}^{d'}$ into the reservoir, and $\mathbf{b} \in \mathbb{R}^N$ is a bias vector. The mapping of the reservoir state onto the control perturbations is given by

$$\mathbf{u}_i = \mathbf{W}\mathbb{O}_{total,i}, \quad (2)$$

where $\mathbf{W} \in \mathbb{R}^{d\times N}$ are the weights learned through ridge regression.

The number of multiplications required by the model is dominated by the sparsity of $\mathbf{A}$, which is characterized by the number of nonzero elements k in each row. The total number of

multiplications contributed by each term are kN from $\mathbf{A}\mathbb{O}_{total,i-1}$, $2Nd'$ from $\mathbf{W}_{in}^{x}\mathbf{X}_i$ and $\mathbf{W}_{in}^{des}\mathbf{Y}_{des,i+m}$, $2N$ from $\alpha\tanh(\dots)$ and $\alpha\mathbb{O}_{total,i-1}$, and dN from $\mathbf{W}\mathbb{O}_{total,i}$. If $\mathbb{O}_{total,i-1}$ is subtracted from $\tanh(\dots)$ before they are multiplied by α, the total number of multiplications are reduced by N, and we assume this best-case scenario. To control the chaotic circuit, Canaday *et al.*[1] uses $k = 3$, $N = 30$, $d' = 2$, and $d = 1$, which requires 270 multiplications, 30 trainable parameters, and 30 evaluations of tanh. Recall that Canaday *et al.*[1] requires a two layer controller to achieve accurate control, which doubles the resources to 540 multiplications, 60 evaluations of tanh, and 60 trainable parameters. This arithmetic is implemented using 32-bit fixed-point arithmetic, but the FPGA has only 18×18-bit multipliers, meaning that additional multipliers are needed for a single multiplication.

Control with next-generation reservoir computing

In our approach, evaluating the control law involves computing the feature vector $\mathbb{O}_{\mathbf{F},i} \in \mathbb{R}^{N-d}$, and then $\mathbf{u}_i$ is computed directly using the control law

$$\mathbf{u}_i = \widehat{\mathbf{W}}_u^{-1}\left[\mathbf{Y}_{des,i+m} - \widehat{\mathbf{W}}_{\mathbf{F}}\mathbb{O}_{\mathbf{F},i} + \mathbf{K}\mathbf{e}_i\right]. \quad (3)$$

The number of multiplications is dominated by the polynomial functions in $\mathbb{O}_{\mathbf{F},i}$, whose number and complexity are determined using system identification techniques,[2,3] and depend on the dynamics of the system under control. For now, we count only the contributions from matrix multiplications in Eq. 3, which are $d(N-d)$ from $\widehat{\mathbf{W}}_{\mathbf{F}}\mathbb{O}_{\mathbf{F},i}$, d^2 from $\mathbf{K}\mathbf{e}_i$, and d^2 from $\widehat{\mathbf{W}}_u^{-1}[\cdots]$.

To control the chaotic circuit, we use $N = 10$, $d = 1$, and $\mathbb{O}_{\mathbf{F},i}$ is given by

$$\begin{aligned}\mathbb{O}_{\mathbf{F},i} = [&u_{1,i-1}, V_{1,i}, V_{1,i-1}, (V_{1,i} - V_{2,i})V_{1,i-1}^2, (V_{1,i-1} - V_{2,i-1})V_{1,i}^2,\\ &(V_{1,i} - V_{2,i})^3, (V_{1,i-1} - V_{2,i-1})^3, V_{2,i}, V_{2,i-1}],\end{aligned} \quad (4)$$

which requires three multiplications for each cubic term, bringing the total number of multiplications to 23. However, we employ a trick to reduce the number of multiplications further by noting that some multiplications are time delayed versions of each other, allowing us to reuse them in future evaluations of the control law. Specifically, $(V_{1,i} - V_{2,i})^3$ becomes $(V_{1,i-1} - V_{2,i-1})^3$, saving three multiplications, and $V_{1,i}^2$ becomes $V_{1,i-1}^2$, saving two multiplications. This brings the total number of multiplications per evaluation of the control law to 18, the number of trainable parameters to 10, and no evaluation of tanh is required. The arithmetic is implemented using 18-bit fixed-point arithmetic, which is well matched to the multipliers on the FPGA and therefore uses even fewer multipliers than the Canaday approach.

Comparing our approach directly to the one-layer controller, we require 15× fewer multiplications, a 3× fewer trainable parameters, and 30 fewer evaluations of tanh. For the two-layer controller, which is more comparable to our approach in terms of performance, we require 30× fewer multiplications, 6× fewer trainable parameters, and 60 fewer evaluations of tanh.

Supplementary Note 2: Time-series trajectories for the origin and random waveform tasks

Supplementary Fig. 1 shows example trajectories during control for the origin task and the random waveform task for a given K. For the origin task, the system is initially far from the desired point but is quickly steered there and remains stable for the rest of the control period with

no visible ringing. It is notable that the control signal required to stabilize the system here is very small, unlike in Canaday et al.[1] For the random waveform task, the system follows the desired trajectory without any visible deviation from the desired signal, despite the great difficulty of this problem.

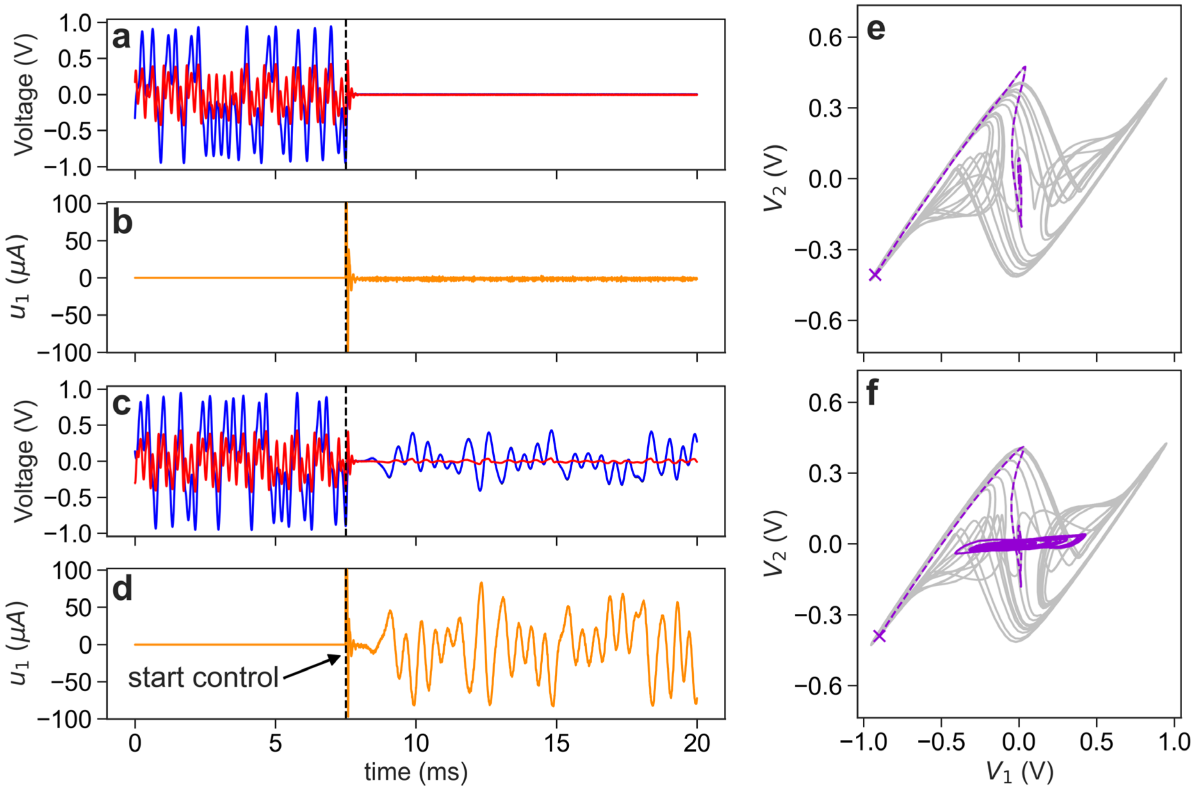

Supplementary Figure 1. Example control trajectories using one-step ahead predictive controller. Time series of controlled system for the origin task (**a**) and the random waveform task (**c**). V_1 (blue) and V_2 (red) display chaotic dynamics before the control begins, and then travel directly over the desired trajectory (solid black). The control signals (**b**) and (**d**) (orange) become nonzero when the control begins. Phase space plots of the dynamics for the origin task (**e**) and the random waveform task (**f**) initially exhibit chaotic behavior (gray), and when the control is switched on (purple ×), the system transitions towards the desired trajectory (dashed purple), and then stabilizes (solid purple).
$K = 0.70$ for all plots.

Supplementary Note 3: Additional performance metrics

In this section, we compare additional performance metrics for the linear, and NG-RC-based controllers with one and two-step ahead prediction. These performance metrics include the control error and size of the control perturbations as a function of the feedback control gain K, and the sensitivity to control loop latency.

The error for each controller type and task as K varies is shown in Supplementary Fig. 2a-c. We see that the domain of control is the largest for both the origin task and the two USS task for the linear and two-step ahead controller. However, the two-step ahead controller has a significantly larger domain of control compared to the linear controller for the random waveform task.

The one-step ahead controller generally has a smaller range of stable K values than the other two controllers, but comparable minimum errors to the two-step ahead controller. We believe that the two-step ahead controller has better performance for multiple reasons. The first is that the latency between measuring the state and applying the control perturbations is a significant fraction of the sample time, so the state of the system one-step ahead is not sufficiently affected by the control perturbation at the current step, and the ML model cannot accurately approximate $\mathbf{W}_u$. The second is that the sensitive dependence to initial conditions present in chaos guarantees that states further in the future are increasingly sensitive to the control perturbation at the current time, so the ML must more accurately approximate $\mathbf{W}_u$ to achieve good prediction performance. Despite the differences between the one-step and two-step ahead controllers, both have significantly lower minimum errors than the linear controller, especially for the random waveform task, showing the robustness of our approach.

The RMS of the control perturbations are shown in Supplementary Fig. 2d-f, and the RMS values that correspond to the minimum control RMSE are shown in Supplementary Table 1. We also show the maximum absolute value of the RMS current after the transient period of 2.5 ms in Supplementary Fig. 3. We see that the size of the control perturbations when the system is stabilized at the origin are larger than when the system is stabilized at either USS at the center of the scrolls for all controllers. However, the linear controller requires larger control perturbations for the random waveform task than the other controllers, demonstrating the effectiveness of our approach.

It is important to characterize how the stability of the system is affected by the control loop latency and the feedback gain K, called the domain of control, which is useful for situations where this latency may drift between the training and control phase. The domain of control for each controller and control task are shown in Supplementary Fig. 2g-i, where the control is classified as stable when the error is below 30 mV. We see that the linear controller has the largest domains of control for the origin task and two USS tasks but fails to maintain stability on the random waveform task as the latency is increased, in contrast to the two-step ahead controller that maintains stability for this task with up to 4 μs of added delay.

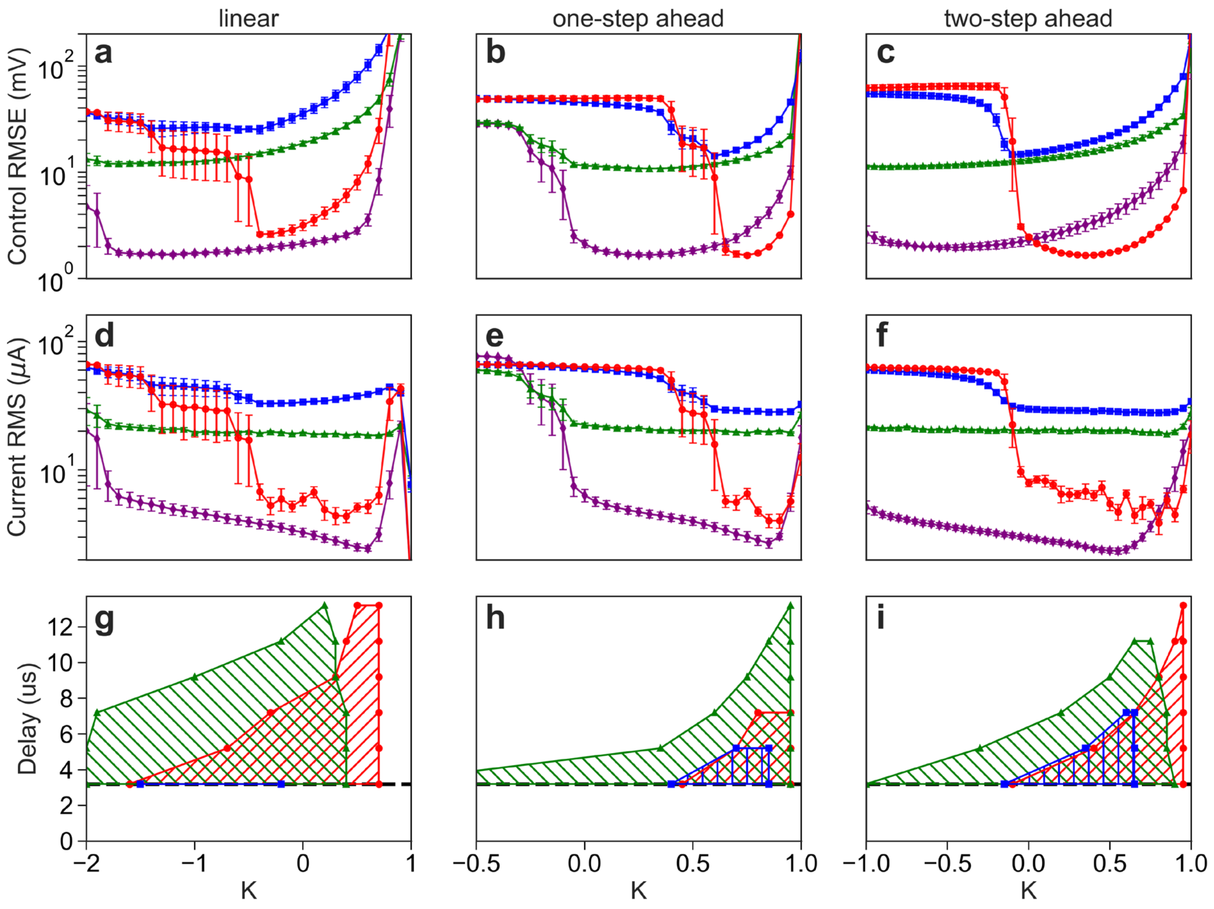

Supplementary Figure 2. Performance of the NG-RC controller. **a-c** RMSE of control, **d-f** RMS of control current, and **g-i** domain of control for the linear, one and two-step ahead NG-RC controllers for the origin task (red circles), two USS task (green triangles), random waveform task (blue squares), and the two USS task excluding transitions (purple diamonds).

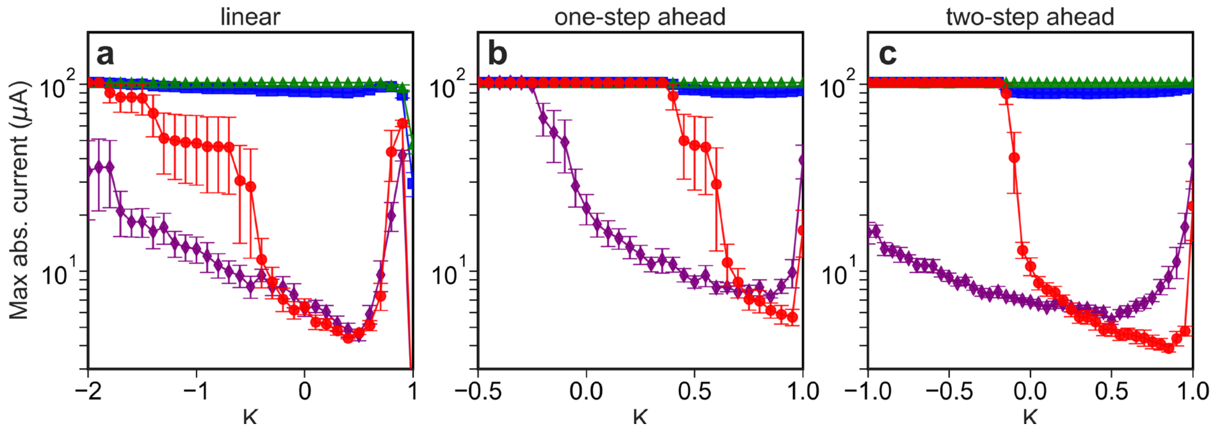

Supplementary Figure 3. Maximum control current. **a-c** Maximum absolute value of the control current after the transient period of 2.5ms for the linear, one and two-step ahead NG-RC controllers for the origin task (red circles), two USS task (green triangles), random waveform task (blue squares), and the two USS task excluding transitions (purple diamonds).

Supplementary Table 1 RMS of control current

Controller type	Origin task	Two USS task	Two USS task (No transients)	Random Waveform Task
Linear	6.79 ± 0.97 uA	21.88 ± 1.21 uA	4.94 ± 0.49 uA	37.17 ± 4.16 uA
one-step ahead	6.54 ± 0.63 uA	20.49 ± 0.47 uA	4.54 ± 0.31 uA	29.75 ± 0.72 uA
two-step ahead	6.84 ± 0.92 uA	21.09 ± 0.27 uA	3.55 ± 0.16 uA	31.24 ± 0.78 uA

The stated values are the RMS of the control current when the control error (RMSE) is minimized with uncertainty derived from the standard error over five trials.

Supplementary Note 4: Comparing control resources

We compare the speed of our controller, the power consumption, and the resource utilization of the FPGA with other nonlinear control studies in Supplementary Table 2. The computational complexity of each of these control tasks is different, so the comparison should only be used as an approximate guide. The energy per inference is calculated using the sampling rate.

Supplementary Table 2 Comparing control resources with other FPGA control work

	This Work	Dedania 2022[4]	Li 2022[5]	Hartley 2014[6]
Task	Chaotic circuit control	Low power PID control	Autonomous Driving	Aircraft control
Model	NG-RC Controller (one-step ahead)	PID Control	Model Predictive Control	Model Predictive Control
Total Power	1384.0 ± 0.7 mW	20 mW	6 W	9.75 W
Dynamic Power	–	–	2.5 W (chip power)	5.469 W
ML Power	5.0 ± 1.4 mW	–	–	–
Solution time	50 ns	–	0.0803 ms (average)	4 ms (max)
Sampling rate	5 μs	1.58 μs	20 ms	0.2 s
Energy per Inference (J)	25.0 ± 7.0 nJ (ML only) (6920.0 ± 3.5 nJ total)	31.54 nJ	50 mJ (chip power) 120 mJ (total)	1.094 J (dynamic) (1.95 J total)
Registers	924 (2 %)	3998 (Logic Cells)(75%)	30059 (Flip flops) (28%)	145526 (49%)
LUT	1497 (3%)	-	47923 (90%)	111132 (7%)
Multipliers	18 (18b × 18b) (13%)	3 (18b × 18b) (37%)	78 (18b × 25b) (35%)	602 (18b × 25b) (78%)
Block Memory	1 (<1%) (9Kb) (9216 total bits)	26 (86%) (4Kb) (104000 total bits)	22 (7%) (18Kb) (~396000 total bits)	142 (34%) (36Kb) (5112000 total bits)

The control resources used for different control algorithms implemented on an FPGA for different control tasks. The "–" denotes that this information is unavailable.

Supplementary Note 5: Elements of W and prediction performance on validation data

For each of the five trials, the weights are learned using different training and validation sets, generated from a different random perturbation. Some of these weights are trained for one-step-ahead prediction ($V_{1,i+1}$) or for two-step-head prediction ($V_{1,i+2}$) in the future. The weights are visualized in Supplementary Fig. 4.

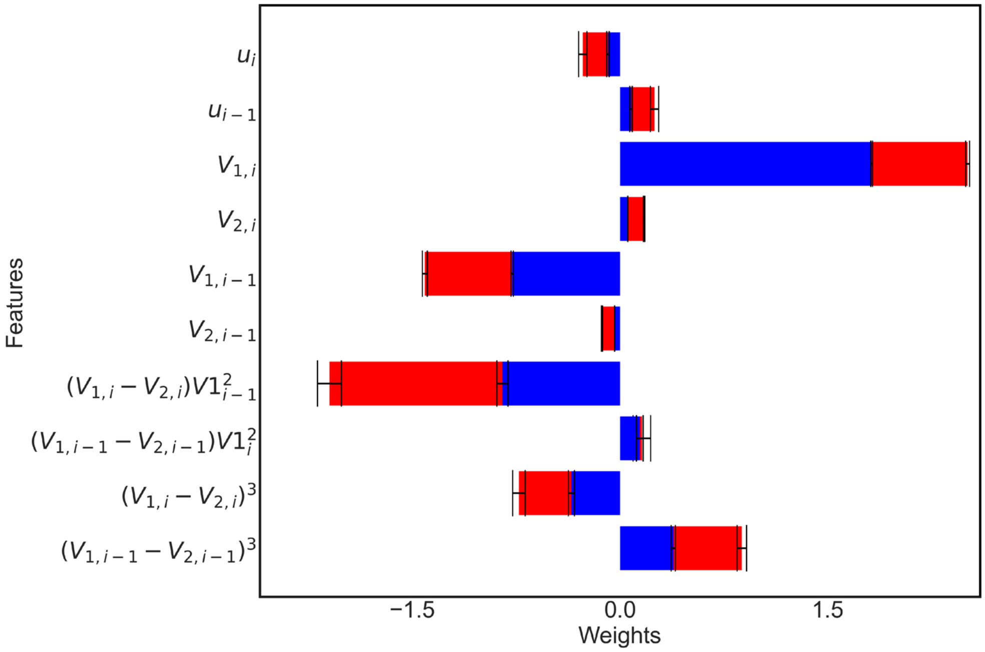

Supplementary Figure 4. Elements of W. (blue) Learned weights for predicting one step in the future (red) and for two steps in the future. The standard deviation of the weights for all five trials divided by the square root of the number of trials is indicated by the black error bars.

For one-step- (two-step-) ahead prediction, the ridge regression parameter giving the minimum prediction error on the validation set, the minimum prediction error, and the fixed-point format are given in Supplementary Table 3 (Supplementary Table 4).

Supplementary Table 3 Prediction performance and Parameters for One-Step-Ahead Prediction

Trial Number	Min. Prediction RMSE (mV)	Ridge Parameter	Fixed point representation for **W**	Fixed point representation for W_u^{-1}
1	2.28	1.29×10^{-7}	Q2.15	Q4.13
2	2.09	2.78×10^{-8}	Q2.15	Q5.12
3	2.15	7.74×10^{-5}	Q2.15	Q4.13
4	2.16	3.59×10^{-5}	Q2.15	Q4.13
5	2.16	2.78×10^{-5}	Q2.15	Q4.13

The minimum prediction RMSE, the optimal ridge parameter, and the fixed-point representation for **W** and $W_u^{-1}$are given for each trial for the one-step ahead prediction model.

Supplementary Table 4 Prediction Performance and Parameters for Two-Step-Ahead Prediction

Trial Number	Min. Prediction RMSE (mV)	Ridge Parameter	Fixed point representation for **W**	Fixed point representation for W_u^{-1}
1	4.58	2.78×10^{-8}	Q2.15	Q2.15
2	3.88	1.00×10^{-5}	Q2.15	Q3.14
3	4.39	7.74×10^{-5}	Q2.15	Q3.14
4	4.23	1.66×10^{-5}	Q2.15	Q2.15
5	3.99	1.29×10^{-5}	Q2.15	Q2.15

The minimum prediction RMSE, the optimal ridge parameter, and the fixed-point representation for **W** and $W_u^{-1}$are given for each trial for the two-step ahead prediction model.

Supplementary Note 6: Prediction performance during control

In this section, we quantify the prediction performance while the controller is active as a function of some of the control parameters such as the feedback gain K, which can be seen for both the one-step- and two-step-ahead prediction in Supplementary Fig. 5. Like the control error presented in Fig. 3 of the main text, the prediction error for all tasks is small for only a fixed range of K values. Notably, this range is larger for predicting two steps in the future, but the minimum RMSE for each task is higher. The minimum prediction RMSE for each task are listed in Supplementary Table 5. This minimum prediction error vs. stability trade-off is an important consideration when designing an NG-RC controller.

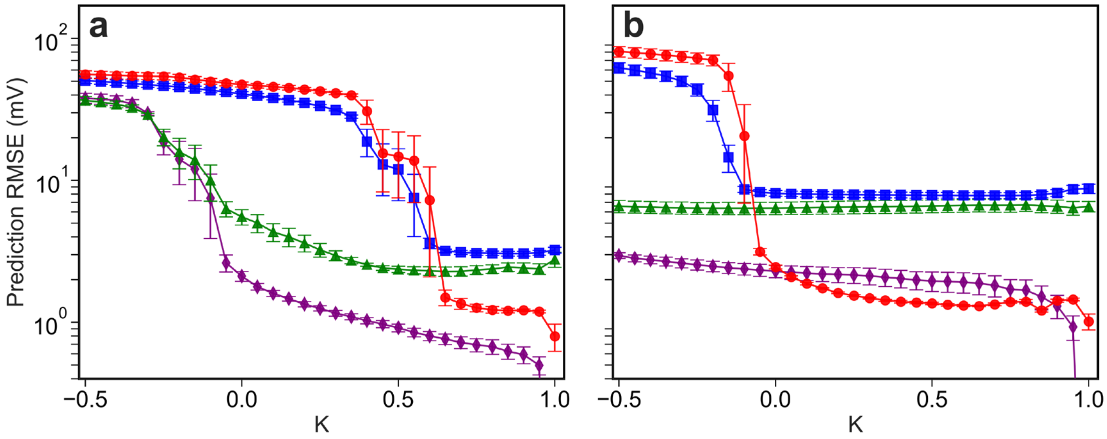

Supplementary Figure 5. Prediction performance during control. RMSE of prediction during control for (**a**) one-step ahead prediction and (**b**) two-step ahead prediction when controlling the system to the phase-space origin (red circles), back-and-forth between two USSs (green triangles), to a random waveform (blue squares), and stabilized at either nonzero USS (purple diamonds) as a function of the control gain K.

Supplementary Table 5 Prediction performance during control

Controller type	Origin task	Two USS task	Two USS task (no transients)	Random Waveform task
one-step ahead	1.19 ± 0.02 mV	2.28 ± 0.17 mV	0.50 ± 0.07 mV	3.04 ± 0.11 mV
two-step ahead	1.21 ± 0.03 mV	6.36 ± 0.63 mV	0.93 ± 0.18 mV	7.82 ± 0.37 mV

The minimum prediction RMSE during the control loop for the one and two-step ahead controllers for each control task, where the error for K = 1 has been excluded because the origin and two USS task (no transients) have significantly lower prediction errors here but control fails.

Supplementary Note 7: Controller latency

In this section, we characterize the minimum latency of the controller because it can affect the domain of control. We find that the latency, defined as the minimum time it takes for an adjustment to exit the controller in response to a change in the input in the absence of the ML model, is a function of the sample rate. We believe the latency is due to the anti-aliasing low-pass filter before the analog-to-digital converter and the serial protocol for transferring data from the analog-to-digital and digital-to-analog converters used to communicate between the FPGA and these off-chip devices. We use the maximum clock rates suggested by the Modular Dual ADC core IP[7] in Quartus Prime 21.1, which range from 1 MHz to 80 MHz depending on the chosen sampling rate, and use a constant 30 MHz clock for the digital-to-analog converter, the maximum allowed by these devices.

We characterized the latency by feeding the analog-to-digital converter with a saw-tooth waveform using the Stanford Research Systems DS345, pass the corresponding digital values directly to the digital-to-analog converter, and measure the output using a Tektronix TDS 210 oscilloscope. The results of this measurement is shown in Supplementary Fig. 6. The minimum

latency is 2.5 μs for the sample rate 1 Msamples/s. For this reason, we use this sample rate in our experiment even though the controller operates at a rate of 200 kHz. We also find that our custom-build voltage-to-current converter adds an additional delay of 0.7 μs independent of sample rate. Additionally, the digital-to-analog converter on the FPGA device has peak to peak settling times of over 2 μs, limiting the rate at which the control perturbations can be switched.

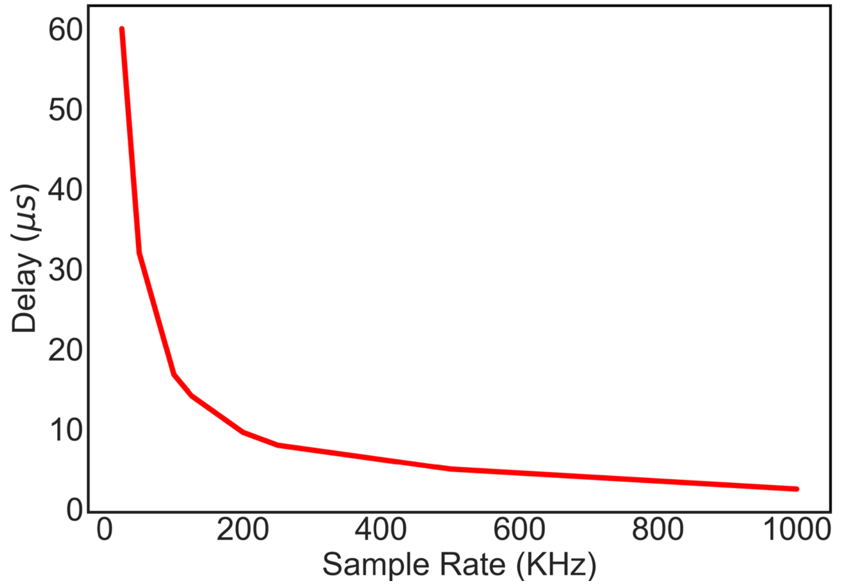

Supplementary Figure 6. Closed loop latency of the controller. Characterization of maximum closed-loop latency of the system as a function of the sampling rate of the analog-to-digital converters.

1. Canaday, D., Pomerance, A. & Gauthier, D. J. Model-free control of dynamical systems with deep reservoir computing. *J. Phys. Complex.* **2**, 035025 (2021).

2. Modular ADC Core Intel® FPGA IP and Modular Dual ADC Core Intel®... *Intel* https://www.intel.com/content/www/us/en/docs/programmable/683596/20-1/and-references.html.

3. Dedania, R. & Jun, S.-W. Very Low Power High-Frequency Floating Point FPGA PID Controller. in *Proceedings of the 12th International Symposium on Highly-Efficient Accelerators and Reconfigurable Technologies* 102–107

4. Li, Y., Li, S. E., Jia, X., Zeng, S. & Wang, Y. FPGA accelerated model predictive control for autonomous driving. *Journal of Intelligent and Connected Vehicles* **5**, 63–71 (2022).

5. Hartley, E. N. *et al.* Predictive Control Using an FPGA With Application to Aircraft Control. *IEEE Transactions on Control Systems Technology* **22**, 1006–1017 (2014).
